# Supplementary material for: microRNA-185 Inhibits SARS-CoV-2 Infection through the Modulation of the Host’s Lipid Microenvironment
Source: Viruses. 2023 Sep 14;15(9):1921. doi: 10.3390/v15091921 (PMC10536008; doi:10.3390/v15091921)
Supplement: Supplementary file 1 [file viruses-15-01921-s001.zip › viruses-2590760-supplementary--update.pdf]

**Supplemental**

# microRNA-185 inhibits SARS-CoV-2 infection through the modulation of the host's lipid microenvironment

Nadine Ahmed[1], Magen E. Francis[2,3], Noreen Ahmed[1], Alyson A. Kelvin[2,3], John Paul Pezacki[1, *]

Affiliations:

[1] Department of Chemistry and Biomolecular Sciences, University of Ottawa, Ottawa, ON, K1N 6N5, Canada

[2] Department of Biochemistry, Microbiology, and Immunology, University of Saskatchewan, Saskatoon, SK, S7N 5A2, Canada

[3] Vaccine and Infectious Disease Organization-International Vaccine Centre (VIDO-InterVac), University of Saskatchewan, Saskatoon, SK, S7N 5E3, Canada

* Corresponding author. E-mail: john.pezacki@uottawa.ca

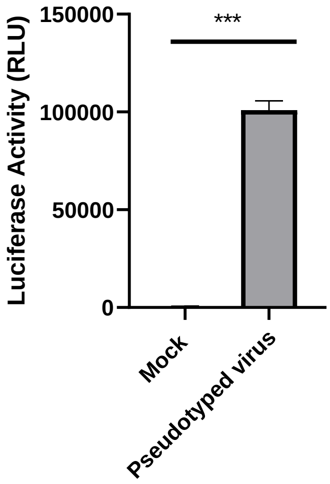


**Figure S1: Confirmation of the functionality of the produced pseudotyped virus.** Mock virus lacking the expression of a functional spike protein results in minimal luciferase activity in recipient cells while virus expression intact spike protein results in increased luciferase activity in recipient/ infected cells. Data presented as Luciferase activity (RLU) Experiments represents three biological replicates. Error bars represent the means ±SEM ,***P<0.001.

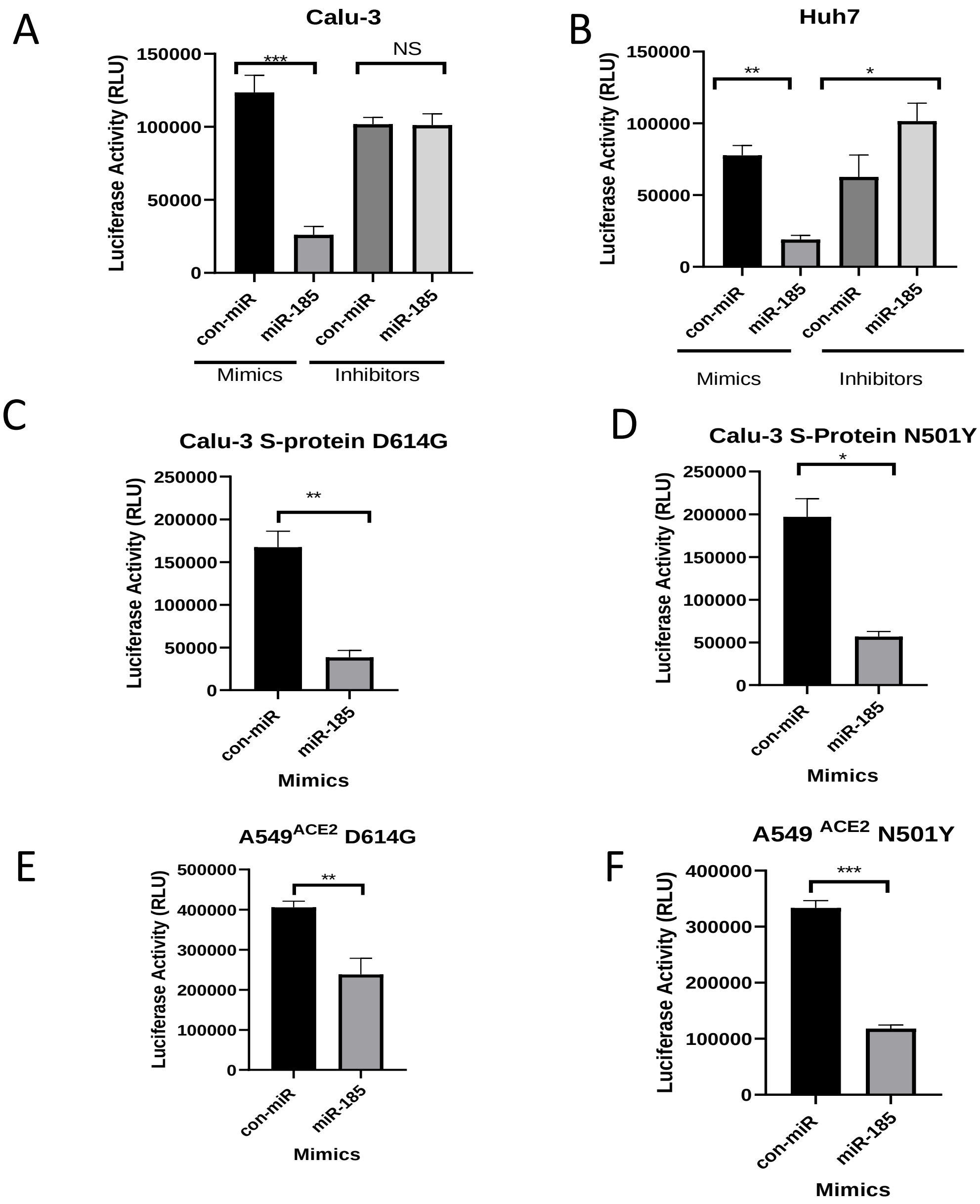


**Figure S2: miR-185 inhibits pseudovirions entry in Huh7 hepatoma cell line and Calu-3 lung carcinoma cell line**. (A) Huh7 cells were forward transfected with miR-185 mimics/inhibitors or con-miR mimics/inhibitors and (B)Calu-3 cells were reverse transfected with miR-185 mimics/inhibitors or con-miR mimics/inhibitors. 24h post transfection, the cells were infected with 100ul of media containing SARS-CoV-2 S pseudotyped virus at an MOI of 2. C) Calu-3 cells infected with S-protein D614G pseudotyped virus D) Calu-3 cells infected with S-protein N501Ypseudotyped virus E) A549 $^{ACE2}$ cells infected with with S-protein D614G pseudotyped virus F) A549 $^{ACE2}$ cells infected with S-protein N501Y pseudotyped virus. 48h post infection, cells were lysed in 1X passive lysis buffer and Luciferase activity was measured using a microplate reader. Data presented as Luciferase activity (RLU) All experiments were read in technical triplicates for at least three biological replicates. Error bars represent the means ±SEM of at least three biological replicates. *P<0.05, **P<0.01, ***P<0.001.

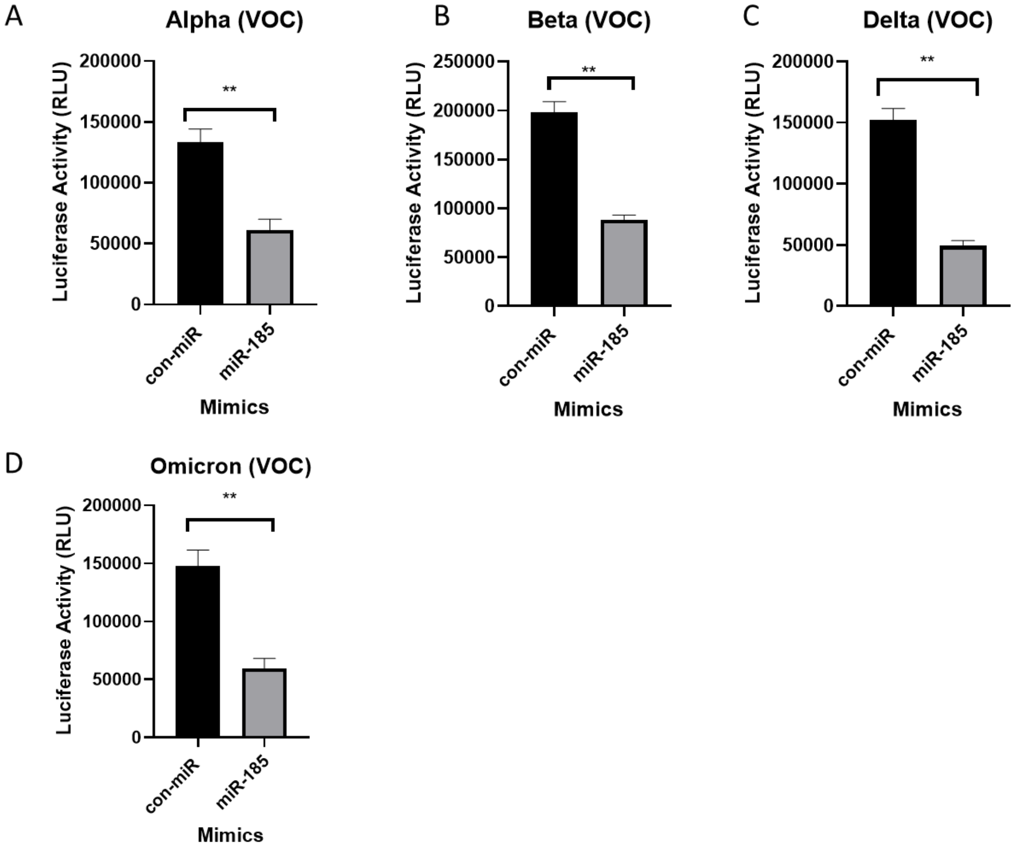


**Figure S3: miR-185 inhibits entry of SARS-CoV-2 alpha, beta, and delta Spike variants pseudotyped virus in cell culture.** Calu-3 cells were transfected with miR-185 mimics or con-miR mimics and 24h post transfection, the cells were infected Sars-CoV-2 S pseudotyped virus (A) Alpha (B)Beta and (C) delta variants of concern (D) Omicron variant of concern. 48h post infection, cells were lysed in 1X passive lysis buffer and Luciferase activity was measured using a microplate reader. Data presented in luciferase activity (RLU). All experiments were read in technical triplicates for at least three biological replicates. Error bars represent the means ±SEM of at least three biological replicates. **P<0.01.

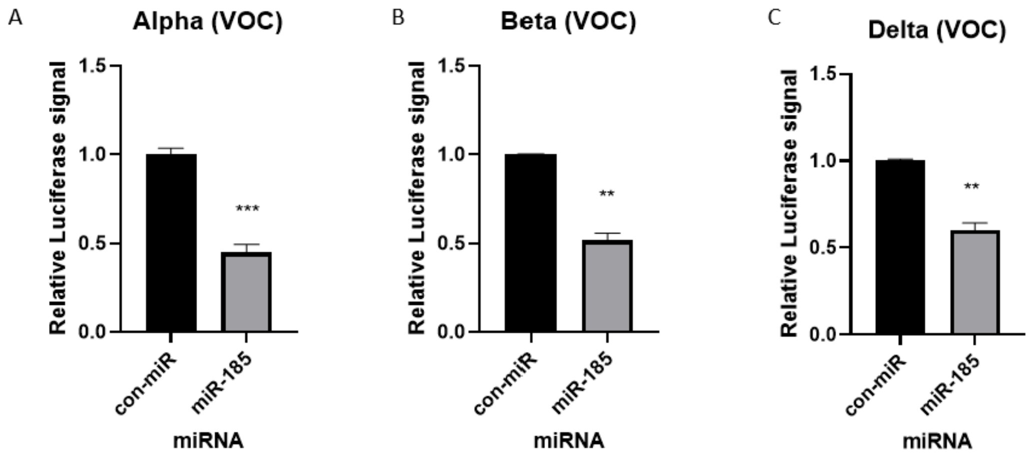


**Figure S4: miR-185 inhibits entry of SARS-CoV-2 Alpha, Beta and Delta variants pseudo-typed virus in cell culture**. Huh7 cells were transfected with miR-185 mimics or con-miR mimics and 24h post transfection, the cells were infected with 100ul of media containing Sars-CoV-2 S pseudo-typed virus (A) Alpha (B)Beta and (C) delta variants of concern. 48h post infection, cells were lysed in 1X passive lysis buffer and Luciferase activity was measured using a microplate reader. All experiments were read in technical triplicates for at least three biological replicates. Error bars represent the means ±SEM of at least three biological replicates. **P<0.01, ***P<0.001

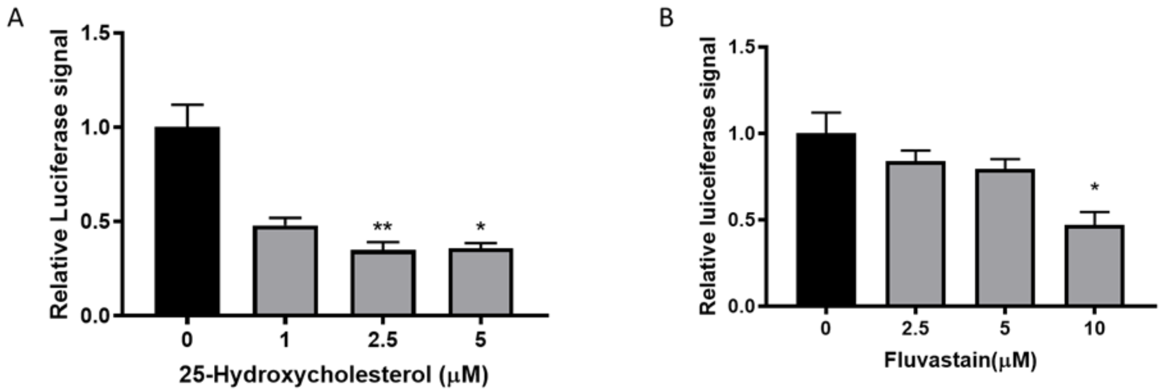


**Figure S5: Inhibitors of SREBP2-regulated signaling inhibit SARS-CoV-2 entry.** Huh7 cells treated with (A)25-Hydroxycholesterol or (B) Fluvastatin at various concentration, cells were infected with 100ul of media containing Sars-CoV-2 S pseudo-typed virus. 48h post infection, cells were lysed in 1X passive lysis buffer and Luciferase activity was measured using a microplate reader. All experiments were read in technical triplicates for at least three biological replicates. Error bars represent the means ±SEM of at least three biological replicates.*$P<0.05$, **$P<0.01$.

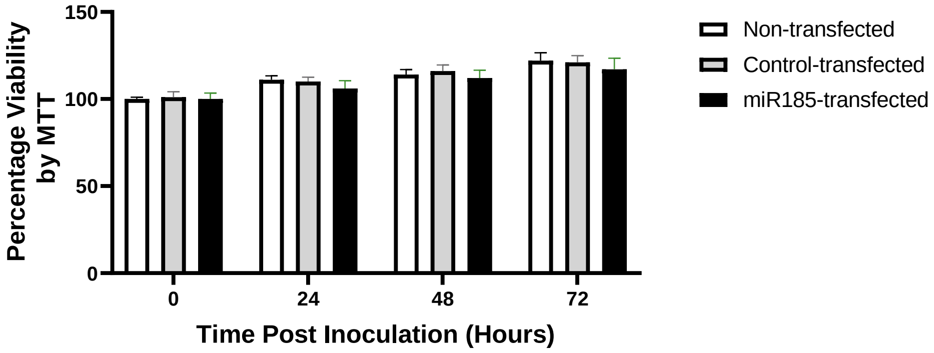


**Figure S6: miRNA transfection does not affect cell viability.** MTT assay confirmed no significant change in cell viability at various timepoints post-transfection of mimic at 100nM concentration in Calu-3 cells.

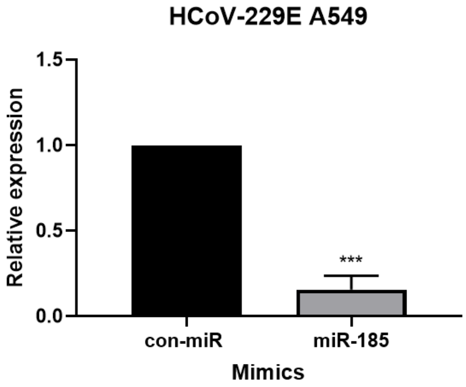


**Figure S7: miR-185 inhibits HCoV-229E pathogenesis in A549 cells**. Huh7 cells were transfected with miR-185 mimics or con-miR mimics.24 h post-transfection, cells were infected with HCoV-229E at an MOI of 0.05. 48h post-infection cells lysed for RNA analysis of intracellular levels of virus. ***P<0.001.

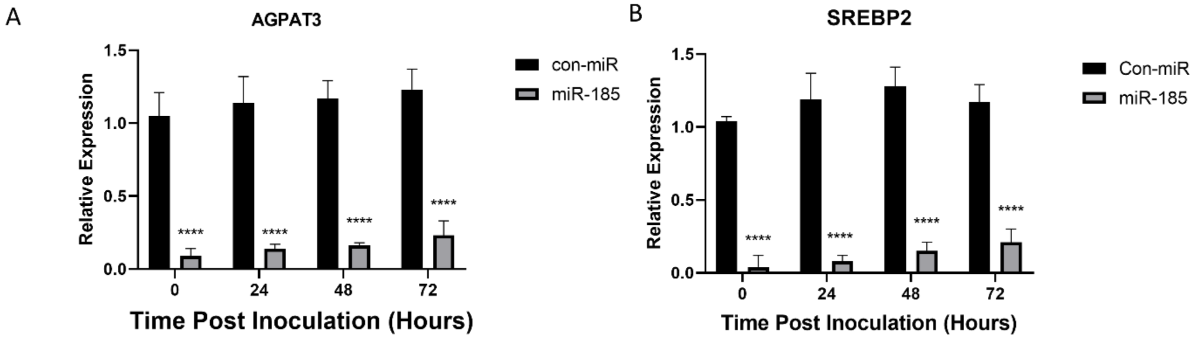


**Figure S8: miR-185 decreases the expression of lipogenic genes during SARS-CoV-2 infection.** Calu-3 cells were transfected with miR-185 mimics or con-miR mimics. Cells were infected with SARS-CoV-2 at various time-points at an MOI of 0.01. Cells were lysed for RNA analysis at 0, 24, 48 and 72h post inoculation. RT-qPCR were performed and the levels of SREBP2 and AGPAT3 were evaluated at each timepoint. ****p<0.0001.

Table S1. List of qPCR primers used in this study

| Oligonucleotide | Sequence |
|---|---|
| RNA18S | FWD: GCGATGCGGCGGCGTTATTC |
| | REV: CAATCTGTCAATCCTGTCCGTGTCC |
| HCoV-229E primers | FWD: TGGCCCCATTAAAAATGTGT |
| | REV: CCTGAACACCTGAAGCAAT |
| SREBP2 | FWD: CTTTGATATACCAGAATGCAG |
| | REV: CAGGCTTTGGACTTGAGGCTG |
| SQLE | FWD: GGCATTGCCACTTTCACCTAT |
| | REV: GGCCTGAGAGAATATCCGAGAAG |
| AGPAT3 | FWD: CTCCAAGGTCCTCGCTAAGAAG |
| | REV: CCGCTTGCAGAACACAATCTC |
| SCARB1 | FWD: TCGCAGGCATTGGACAAACT |
| | REV: CTCCTTATCCTTTGAGCCCTTTT |
| PPARg | FWD: AGCCTGCGAAAGCCTTTTGG |
| | REV: GGCTTCACATTCAGCAAACCTGG |
| ACE2 | FWD: CGAAGCCGAAGACCTGTTCTA |
| | REV: GGGCAAGTGTGGACTGTTCC |

Table S2. Antibodies used in this study

| Primary Antibody | Primary Dilution | Provider | Catalog number |
|---|---|---|---|
| B-tubulin | 1:5000 | Abcam | 610621 |
| LSS | 1:1000 | ProteinTech | 13715-1-AP |
| SREBP2 | 1:250 | BD Pharmingen | 557037 |
| Spike | 1:2000 | GeneTEX | GTX632604 |
